# Radiomic and radiogenomic modeling for radiotherapy: strategies, pitfalls, and challenges

**DOI:** 10.1117/1.JMI.8.3.031902

**Published:** 2021-03-23

**Authors:** James T. T. Coates, Giacomo Pirovano, Issam El Naqa

**Affiliations:** a Massachusetts General Hospital & Harvard Medical School, Center for Cancer Research, Boston, Massachusetts, United States; b Memorial Sloan Kettering Cancer Center, Department of Radiology, New York, New York, United States; c Moffitt Cancer Center and Research Institute, Department of Machine Learning, Tampa, Florida, United States

**Keywords:** radiogenomics, radiomics, radiotherapy, predictive modeling, outcomes

## Abstract

The power of predictive modeling for radiotherapy outcomes has historically been limited by an inability to adequately capture patient-specific variabilities; however, next-generation platforms together with imaging technologies and powerful bioinformatic tools have facilitated strategies and provided optimism. Integrating clinical, biological, imaging, and treatment-specific data for more accurate prediction of tumor control probabilities or risk of radiation-induced side effects are high-dimensional problems whose solutions could have widespread benefits to a diverse patient population—we discuss technical approaches toward this objective. Increasing interest in the above is specifically reflected by the emergence of two nascent fields, which are distinct but complementary: radiogenomics, which broadly seeks to integrate biological risk factors together with treatment and diagnostic information to generate individualized patient risk profiles, and radiomics, which further leverages large-scale imaging correlates and extracted features for the same purpose. We review classical analytical and data-driven approaches for outcomes prediction that serve as antecedents to both radiomic and radiogenomic strategies. Discussion then focuses on uses of conventional and deep machine learning in radiomics. We further consider promising strategies for the harmonization of high-dimensional, heterogeneous multiomics datasets (panomics) and techniques for nonparametric validation of best-fit models. Strategies to overcome common pitfalls that are unique to data-intensive radiomics are also discussed.

## 1 Introduction

Radiotherapy is received by approximately half of all cancer patients.^1^ The development of predictive models for determining which patients are likely to benefit from radiotherapy and which are at risk of incurring aberrant toxicities could, therefore, provide benefit to a large population. Early efforts to capture radiation-induced side effects or assign a probability for achieving local control consisted principally of dose–volume effect correlations^2^^,^^3^—these approaches yielded some success with classical treatment modalities, but are now recognized to be intrinsically limited due to patient heterogeneity at the biological level.^4^^–^^6^ Two distinct but complementary strategies have emerged in recent years to overcome this limitation: (i) the integration of patient-specific biological risk factors into dose–volume-based outcome models, which we refer to herein as radiogenomics,^7^ and (ii) the integration of imaging correlates together with treatment-related and biological data for outcomes prediction, radiomics.^8^ We note that an alternative definition of radiogenomics exists in literature entailing the exclusive use of imaging correlates and genomic data;^9^ however, given our focus on radiotherapy outcome modeling, we forgo this definition in preference of the above. Taken together, there is renewed interest in the development of personalized radiotherapy treatment plans and, eventually, their integration into automated treatment planning systems.^10^^,^^11^

We review radiogenomic and radiomic modeling strategies herein first by discussing the types of data and derivative metrics (correlates) commonly used, including next-generation datasets. Classes of outcomes associated with either tumor control or normal tissue toxicity are then reviewed. We consider conventional dose–volume-based approaches for outcomes modeling, which sets the stage for discussion of radiogenomic and radiomic modeling approaches and furthermore highlight techniques for the augmentation of classical models allowing them to incorporate biological and/or clinical risk factors. Both conventional and deep learning strategies are reviewed in the context of radiomics. Given the emergence of next-generation technologies and large datasets obtained from unique sources, we discuss potential outcome modeling strategies using the integration of heterogeneous and high-dimensional multiomics datasets (panomics).^12^ As they are critical for any modeling approach, we conclude by discussing common pitfalls for data-intensive radiomics or panomics and validation methods that can be used to maximize reproducibility and robustness of best-fit models.

## 2 Definition of Risk

Predicting outcomes for radiotherapy necessitates a definitive understanding of risk (that is to be predicted). In the case of radiotherapy, there are two main categories:^6^ (i) tumor control probability (TCP) and (ii) normal tissue complication probability (NTCP). TCP refers to the probability of success for the treatment procedure defined over a time period. Ideally, TCP models would also take into account the possibility of recurrence at longer times after treatment, but data for this can be sparse and furthermore depend on factors that significantly increase model complexity.^5^ NTCP by contrast defines the risk of specific aberrant toxicities induced as a consequence of ionizing radiation and are generally attributed to damage of healthy tissues.^6^ It has become recognized that better performing NTCP strategies necessitate the inclusion of data beyond dose–volume metrics, such as the functional statuses of genes that significantly modulate normal repair processes, or the use of other biologically relevant data, to better capture interpatient variabilities.^13^^,^^14^

## 3 Radiotherapy Outcomes (Endpoints)

Ionizing radiation induces effects that span nanoseconds (free radical production) to years after delivery (late side effects). Upon delivery, free radicals diffuse and induce a complex cascade of molecular and cellular processes that may only become manifest and clinically detectable days, weeks, months, or years later.^15^^,^^16^ Every effort is made to spatially target radiation to cancer cells; however, normal cells and critical structures adjacent to target volumes unavoidably receive a portion of the therapeutic dose.^17^ Herein, we refer to toxicity from radiotherapy as dose-limiting and this may include acute effects or late adverse events as detailed below.

### 3.1 Early Side Effects

Acute side effects from radiation therapy are often transient, self-limiting events that can typically resolve within a few weeks post-treatment and do not induce severe or long-term morbidity, although this might vary patient-by-patient. An example is the acute inflammation or ulceration of mucosal membranes inducing mucositis in head and neck cancer treatment.^18^ Notably, a difficulty in assessing early side effects is the interpretation of data from independent sources that may use different grading schemas or endpoints.^19^

### 3.2 Late Toxicity Events

Late radiation-induced damage to normal tissue occurs empirically >90 days after completion of radiotherapy. Late toxicities can often be difficult to assess, as no quantitative physiological evidence exists or can readily be obtained, and include mild, moderate, severe, and life-threatening morbidities, sometimes requiring additional intervention to be mitigated. Grading schemes have been developed to classify such morbidity by physicians assigning integer values to the induced side effects. These could be self-scoring questionnaires or grades assigned by an attending physician. The correlation between different scoring schemes using a single set of data can be explored utilizing various machine learning (MI) techniques that rely on the accuracy of outcome measures.^20^^–^^22^

### 3.3 Local Control Endpoints

Reporting of local control is usually specific to a given site. For instance, in prostate cancer, clinical studies typically report local control according to the ASTRO-RTOG Phoenix definition of biochemical failure.^23^ Nondichotomized metrics such as prostate-specific antigen (PSA) doubling time or PSA scores over time could also be used as endpoints in modeling frameworks if continuous outcomes measures are required but may be less reliable.^24^

## 4 Modeling Workflow

**Fig. 1:**
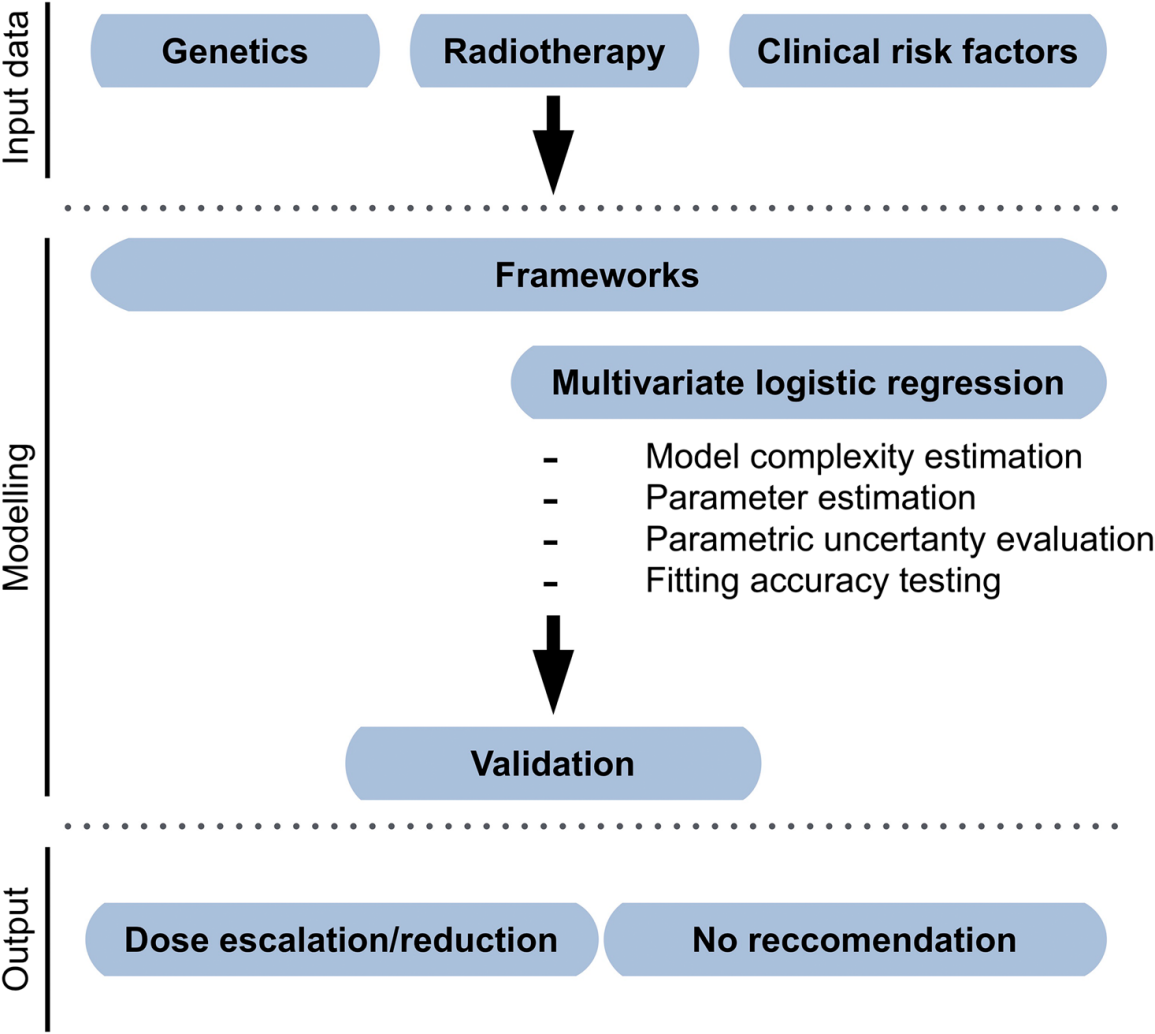
Example of radiogenomic modeling framework. Clinical, dosimetric, and biological data are regressed using a logistic transformation. Internal resampling is used as internal validation. A recommendation of dose escalation, de-escalation, or no recommendation can be generated according to desired thresholds for risk.

**Fig. 2:**
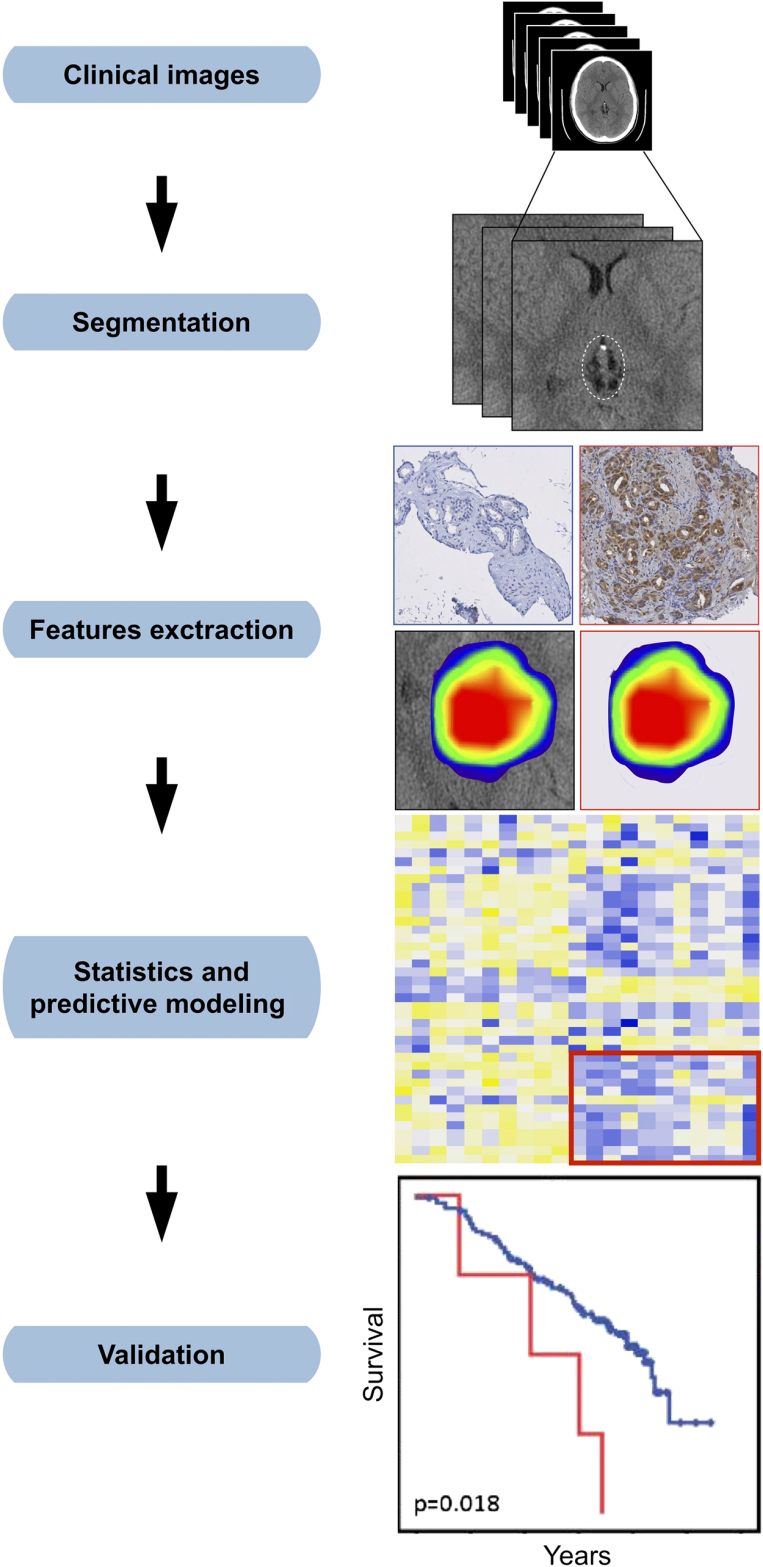
Image processing steps for radiomics modeling. Extracted features from a variety of clinical imaging modalities can be used for radiomics, but typically include one or more of MRI, CT, and positron emission tomography (PET). Segmentation entails the delineation of ROI, which could make use of multimodality acquisition. Features are extracted and used as input data for modeling. Predictive modeling is then performed using a selected framework or strategy before validation and assessment of model generalizability.

The overarching objective of both radiogenomics and radiomics is to prospectively assess the suitability of a patient for a particular treatment regimen; however, the two approaches have unique requirements. Radiogenomics leverages genetic and/or other biological information for improving the prediction of dose–volume-based models, such as in Fig. 1. Radiomics, however, further requires the processing of imaging records to derive metrics that can be used as input data for modeling (Fig. 2). Herein, we consider radiomics to include all aspects of radiogenomics, but strive to discriminate between the two strategies when required as the introduction of imaging features restricts suitable modeling approaches.

### 4.1 Retrieval of Nonimaging Input Data

Dose and volume metrics can be quickly extracted from dose–volume histograms at the treatment planning stage. Clinical data are important in outcomes modeling and can be parameterized as a covariate of interest.^13^ An example where clinical information may be key is the use of anticoagulants and the reporting of aberrant bleeding, for instance. The spatial dose–volume histogram (z-DVH) can also be used to compare spatial treatment planning information with outcomes and many resources exist for their derivation.^25^^,^^26^ Biological variables generally refer to single-nucleotide polymorphisms (SNPs) but can also include copy number variations (CNVs)—these mutations can be quantified by genotyping/sequencing as per our previous work and input into frameworks as dichotomous factors.^27^ We discuss each of these in more detail in the next major section with the exception of datasets that consist of next-generation sequencing (NGS), or similar high-throughput approaches (high-dimensional datasets), which are discussed as panomics strategies later.

### 4.2 Modeling Framework

NTCP or TCP modeling can be divided into two overarching methods: analytical or data-driven. Analytical approaches are based on simplified versions of the underpinning biochemical effects that treatment has on different tissues—analytical models are, therefore, also referred to as quasianalytical due to their recognized limitations discussed in detail elsewhere.^6^ In contrast, data-driven models, or multimetric, are phenomenological in nature such that modeling frameworks based on them will seek to find the best performing model to capture the underlying effects regardless of the constituents of the model itself (unbiased).^28^ Each of these methods is described in detail in Sec. 6.

### 4.3 Model Validation

The importance of crafting a modeling framework itself is critical, but internal performance evaluation prior to testing on unseen data has increasingly become recognized as an important step in predictive modeling—this is especially the case for more complex models whereby the opportunities for overfitting a particular dataset is significant.

There are several strategies that can be applied to quickly ascertain whether the results of a modeling exercise are suitable to proceed to the next steps or whether the framework requires fine-tuning. Most commonly used are nonparametric statistical measures, such as the Spearman’s rank correlation (rs, or ρ).^28^ Used in combination with Chi-squared ($\chi^{2}$) statistical analyses, the degree of fit between each model parameter and the observed outcomes can be assessed without a need for increased computational power. As we discuss, information theory approaches and, more commonly, resampling techniques (e.g., cross-validation through jack-knifing or bootstrapping) can be used to evaluate a model’s complexity relative to its fit to assess the performance of the model on unseen data (generalizability).^29^

### 4.4 Image Processing for Radiomics

Radiomics approaches typically include steps similar to radiogenomic modeling (data retrieval, modeling, validation), but with added caveats relating to image acquisition, tumor segmentation, and feature extraction before predictive modeling and model validation (Fig. 2).^5^^,^^9^ Because of the amount of data generated in radiomics, the most common approach for modeling is MI. Moreover, in contrast to dose–volume metric modeling, no analytical associations of imaging correlates or derivatives and clinical endpoints currently exist, thus restricting radiomics to data-driven techniques.

#### 4.4.1 Image acquisition

Various imaging modalities such as computed tomography (CT), magnetic resonance imaging (MRI), and 18F-fluorodeoxyglucose-positron emission tomography (FDG-PET) are used in the clinics to provide visualize and evaluate the underlying anatomical or physiological properties, which vary intrapatient and interpatient. Unfortunately, there is often a lack of standard methods for image investigation and acquisition, which can represent a barrier to the pan-applied radiomics approach.^30^ From the radiomics point of view, it is crucial to have homogeneous data (albeit through preprocessing or standardization during acquisition) when dealing with multicenter sources, as it is a necessity for validation. Recent developments in high-performing computing can help by mitigating variability and with harmonizing data.^31^^,^^32^

#### 4.4.2 Tumor segmentation

Segmentation is the step of distinguishing the tumor area from the acquired images. Treatment plans in radiotherapy patients already provide a tumor delineation that can be used retrospectively in the radiomic analysis. However, this human-based approach can also present variations among different oncologists or institutes. Radiomic modeling allows testing models for such variations by introducing tumor region perturbations.^9^ An alternative to such an approach would be the use of segmentation algorithms, e.g., deep-learning techniques, which can learn the features along with the classification task.^33^^–^^36^

#### 4.4.3 Feature extraction

To fully characterize the tumor area, both semantic and agnostic radiomic features can be used. The first is existing radiology tumor descriptors. The latter is purely computational features, which can describe shape, density, and texture to define intertumoral.

## 5 Input Data for Modeling Frameworks

A plethora of metrics exist for use as inputs to modeling frameworks. These can be of different natures and can add complexity to the model to virtually an endless number of degrees. However, larger datasets can introduce high amounts of complexity, which can make it challenging to produce an accurate model. The main data types are described below.

### 5.1 Physical

Outcome modeling starts from dose–volume metrics, i.e., dose to a given volume or volume of tissue receiving a particular dose. Already at the stage of treatment planning, it is possible to derive such parameters. Other variations, such as physiological changes and changes in tumor composition or anatomy, may take place during treatment. For this reason, often, the delivered dose may not necessarily reflect the actual biologically absorbed dose. Intrafractional computed tomography (CT) scan changes could be included to improve predictive accuracy.^37^

The equivalent uniform dose (EUD) can be used to describe inhomogeneous dose distributions [Eq. (1)]^38^ and an extension of the EUD for include normal tissue dose is the generalized EUD (gEUD) [Eq. (2)]

$$\mathrm{EUD}={\left(\underset{i}{\sum }v_{i}D_{i}^{1/a}\right)}^{a},$$ (1)

$$\mathrm{gEUD}={\left(\underset{i}{\sum }v_{i}D_{i}^{a}\right)}^{1/a},$$ (2)

where $v_{i}$ represents fractional volume for the tissue exposed to a dose $D_{i}$, and the parameter a refers to the volume effect of a chosen tissue type. These two metrics serve as excellent tools to summarize dose–volume distributions.

### 5.2 Clinical

Other than demographic data, clinical data can impact significantly the outcome modeling and should be parameterized and used to define the covariates. Anything that might have an impact on patients’ health can be incorporated and can potentially extend the model to any degree of complexity.

Covariates of interest can be extracted from clinical data. For example, follow-up therapies such as anticoagulants or androgen deprivation therapy can induce effects that are difficult to distinguish from late toxicities.

### 5.3 Spatial

When comparing spatial treatment planning information with clinical outcomes, z-DVHs can be used.^25^^,^^26^^,^^39^^,^^40^ This approach carries the advantage of incorporating spatial information about the physical location of dose extremes and does not rely solely on volume-averages (or gEUD) approaches. This reduces the risks of undervaluing the contribution of hot or cold spots.

### 5.4 Biological

The most popular class of biological variable found in literature today is related to genetic mutations, but frameworks can be augmented to include epigenetic and transcriptomic data—especially pertinent to long-term radiotherapy outcomes.^41^^,^^42^

#### 5.4.1 Genetic variables

Radiotherapy efficacy and toxicity is a fine balance regulated by a number of biological variables and mechanisms. With the aim of maximizing prospective classification performance, modeling frameworks will need to incorporate several genetic variables. Known variables of biological data are related to genetic mutations. The role of epigenetics and transcript expression levels in long-term radiotherapy outcomes should also be taken into account.^43^^–^^46^ Consider even that recent hypotheses have been raised about the role of the microbiota as a key player in cancer therapy response.^47^

For data-driven modeling, genetic parameters can be included as independent variables and regressed alongside other factors such as clinical risk and dose–volume metrics. For analytical models, genetic parameters are integrated in different ways; dose-modifying factors (DMFs) obtained from clinical risk factors can be used to stratify standard analytical models and generate “mixed” data-type model.^48^ To maximize prospective classification performance, this approach can be expanded to include SNPs resulting in significantly improved classification performance.^49^ This approach can be further extended using clinical risk factors for logit and EUD models.^50^ Further to SNPs, CNVs can represent an effector of radiotherapy outcomes, and therefore, be included.^27^^,^^51^

##### Augmenting Dose–Volume Models with Genetic Variables

Genetic features represent independent variables in data-driven modeling together with dose–volume metrics and clinical risk factors. DMFs obtained from risk factors can be used for standard analytical model stratification to generate mixed data-type models.^52^ This approach can be further developed to include clinical risk factors for logit and EUD modeling.^50^

#### 5.4.2 Epigenetics and transcriptomics

Additional biological variables include epigenetic alterations, with the extra complications of these including therapy intervention as well as an effector of the epigenetic landscape.^53^ An epigenetic code is under development^54^ and may facilitate the ability to perform and interpret the epigenome-wide association studies results and provide a new class of input data for outcome models.^55^

Even with supervised learning algorithms to preprocess the data, the number of messenger ribonucleic acid (mRNA) transcripts from a single microarray experiment can be challenging and often requires large-scale validations. In such a big-data environment, machine-learning techniques in artificial intelligence (AI) have the ability to process highly structured, high-dimensional data, and control for over- and underfitting at the same time.

#### 5.4.3 Next-generation data

In the big-data era, numerous methods used to quantify large numbers of biological factors have been pioneered and introduced into mainstream biology research within the last decade. These technologies include well-characterized microarrays and proteomic analysis technologies that can quantify the levels of expression of up to tens of thousands of mRNA transcripts or proteins in a single sample.

After generating large quantities of data, high-throughput modeling frameworks can be used that are able to deal with large numbers of variables.^56^ This approach has been used successfully in clinical oncology to stratify tumor phenotypes and estimate prognoses to help guide optimal therapeutic regimens.^57^

### 5.5 Imaging

There are a variety of textures that can be extracted from medical images and used as correlates.^58^^–^^61^ Perhaps the most common are qualitative features (semantic) that otherwise can be used to identify potential lesions. In contrast, quantitative features are algorithmically derived from images. These two classes of features can vary widely in complexity; however, both produce metrics that describe the shape and intensity of the voxel histogram and the spatial arrangement of the voxels themselves, i.e., textures.^5^ Notably, features can be directly extracted from the images or extracted after transforming the raw imaging data.

There are several types of quantitative features that can be derived: shape features and statistics that are first order, second order, or higher order.

#### 5.5.1 Shape features

These delineate the geometry of the regions of interest (ROI) in addition to other related properties such as volume, diameter, surface area, and sphericity. First-order statistics are features agnostic to spatial information and describe individual voxel values. Considering a summation of individual voxels represented as a histogram these might include, for instance, mean, min, max, median, skew, and kurtosis.

#### 5.5.2 First-, second-, and higher-order statistics

These can also be referred to as textural features since they pertain distinctly to the relationship of voxels to one another rather than the individual voxels themselves.^62^ They, therefore, provide information on the geometry or arrangement of voxels for use in describing intratumoral heterogeneity. Deriving second-order statistics can be done from the gray-level co-occurrence matrix^63^ or the gray-level run-length matrix,^64^ which both describe voxel intensity distributions across fixed directions. Beyond first- or second-order statistics, there are higher-order statistics, which overarchingly group features that are extracted after filtering or transforming images. Preprocessing of images may involve simple denoising or more complex transformations such as wavelet^65^ or Laplacian.^66^

Given the above, it, therefore, becomes apparent that there exist many correlates that can be derived from any one single image. Importantly, however, a majority of extracted features will be redundant or interdependent.^67^ As such, it is important to first identify endpoints that are clinically relevant to inform what correlates may be most relevant.

Radiomics does not stop here, however, as it may furthermore encompasses the use of -omics data to augment the predictive value of such imaging correlates. Together, the integration of NGS data together with imaging correlates should provide a better opportunity to capture underlying biophysical effects of radiation therapy in an unbiased way. Techniques for the integration of -omics data with imaging correlates typically entail the use of MI approaches (see Sec. 6.4). Indeed, applications of panomics in the clinic are expected to be transformative.^68^^,^^69^

## 6 Modeling Strategies

### 6.1 Dose–Volume Approaches

Outcome modeling typically consists of the evaluation of dose to a given volume of tissue (dose–volume metrics). These parameters already can be used at the treatment planning stage and extracted from dose–volume histograms. However, because physiological changes are expected to occur during treatment as therapeutic effect and as morbidity, the delivered dose does not necessarily reflect biologically absorbed dose. To induce an improved predictive accuracy, intrafractional CT scan changes can be incorporated.^37^ Typically, to summarize dose distributions across volumes, inhomogeneous dose distributions are modeled as EUD or gEUD if considering the normal tissue of interest.^38^^,^^70^

#### 6.1.1 Analytical

Models of the analytical class, also known as mechanistic models, are based on theoretical mechanisms of action of radiobiological intervention. They include growing-in-complexity levels of mechanistic insight into a specific mechanism by which radiotherapy outcomes become evident.

##### Linear-quadratic formalisms

Nearly all analytical dose–volume TCP models make use of the well-known linear-quadratic (LQ) formulation for predicting cell-kill:

$$\mathrm{SF}=e^{-\alpha D-\beta D^{2}},$$ (3)

where SF is the surviving fraction for a given radiation fraction of size D. The coefficients α and β relate to tissue-specificity of single- and dual-track cellular deactivation. More commonly, these coefficients are reported as a ratio (α/β). When formulated as per Eq. (3), the LQ model has been shown to prove valuable for predicting cellular responses *in vitro* and with particular qualities of radiation only. Consequently, reformulations to the canonical LQ model have been done extensively to take into account multifraction regimens, repopulation kinetics, high linear energy transfer radiation, increased dose rates, and stereotactic-body radiotherapy delivery, just to name a few.^6^ Previous works can readily be consulted for further details of LQ formulations^71^ but have, to a degree, fallen into disuse on account of data-driven approaches but also because normal tissue toxicities are dose-limiting in modern treatment regimens.

##### Lyman–Kutcher–Burman

The Lyman–Kutcher–Burman (LKB) model is the most used analytical method for predicting NTCP [Eqs. (4) and (5)]

$$\mathrm{NTCP}(D,D_{50},m)=\frac{1}{\sqrt{2\pi }}\int_{-\infty }^{t}e^{\left(\frac{-u^{2}}{2}\right)}du,$$ (4)

$$t=\frac{D-TD_{50}(v)}{m\cdot TD_{50}(v)}\;\text{and}\;TD_{50}(v)=\;TD_{50}(1)\times v^{-n},$$ (5)

where m is the slope of the best-fit NTCP sigmoid, $TD_{50}(1)$ is the dose at which NTCP=50% for a specific endpoint, and $TD_{50}(v)$ is the tolerance dose for a given partial volume with tissue-specific volume exponent n. The canonical LKB model allows to stratify patient risk based in their EUD relative to the dose at which NTCP is 50% for a specific endpoint.

The canonical LKB model has been shown to be fairly consistent toward different data types and to be able to provide reliable first-order calculations. These calculations are straightforward and the model is able to obey the boundary conditions without additional constraints being needed.^72^

Despite its success in modeling some of the risk factors associated with radiotherapy, the LKB model still has room for improvement, for example, in the case of radiation pneumonitis (RP). Clinical evidence can show that almost all patients undergoing radiotherapy have an RP risk of <50%. However, it is likely that this is a strong underestimation of the actual risk of RP.^49^

##### Binomial Models

This class of analytical NTCP model arises by modeling tissues as composed of function subunits (FSUs). The arrangement of the FSUs in a given tissue is based partially on physiological modeling and partly on empirical evidence. The classical example is that of the gastrointestinal tract, wherein the tissue is considered to have a more parallel architecture such that irradiation of one subsection of the tract does not directly compromise the integrity of the crypt cells that received a subtherapeutic dose.^73^ Conversely, the spinal cord is more serial in nature whereby irradiation of one section may compromise inferior compartments.^4^ In this way, differences in response according to architecture can better be captured. The most often used formalization is the critical volume (CV) model

$$P_{i}=(\begin{array}{c} N\\ t \end{array})P_{\mathrm{FSU}}^{t}{\left(1-P_{\mathrm{FSU}}\right)}^{N-t},$$ (6)

where the first term of the right-hand side of the equation is the binomial coefficient for N and t, $P_{\mathrm{FSU}}$ is the probability that t of N subunits will be deactivated by ionizing radiation, N is the total number of subunits, and t is the number deactivated. The use of the term CV arises since some tissues can be deactivated by a volumetric effect (as discussed). Using binomial statistics, which arise naturally from the consideration of radiation deactivation and the underlying tissue as more parallel or more serial, then the probability that M subunits will be deactivated can be calculated

$$P=\overset{N}{\underset{t=M+1}{\sum }}P_{t}=\overset{N}{\underset{t=M+1}{\sum }}(\begin{array}{c} N\\ t \end{array})P_{\mathrm{FSU}}^{t}{\left(1-P_{\mathrm{FSU}}\right)}^{N-t}.$$ (7)

Importantly, the CV model and the FSU concept do not preclude tissues that may exhibit both parallel and serial effects to one degree or another. These arrangements of FSUs are termed complex, but may similarly be modeled using Eqs. (6) and (7).

Notably, the CV model has been shown to yield the LKB model with specific factors set as constant, suggesting the latter may be a specific case of the former.^73^

Before extensive use of data-driven modeling, CV models and the FSU concept were widely applied. For instance, applications in prostate cancer yielded some success to predict late toxicities, but were never widely clinically adopted. Indeed, while better incorporating tissue architecture, the CV model does not directly consider biological mutations that predispose individual patients to aberrant toxicities. Nor can CV models readily take advantage of NGS data.

#### 6.1.2 Data-driven

Data-driven approaches, also known as phenomenological or statistical techniques, are based on empirical observations and are typically more robust than the analytical approach counterparts. It is not uncommon for this type of modeling to require preprocessing of data as the number of variables to take into account can be large.

##### Regression-based approaches

To fit TCP or NTCP, it is possible to use functions such as logit

$$\pi (X_{i})=\Phi [g(X_{i})],$$ (8)

and probit

$$\pi (X_{i})=\frac{e^{g(X_{i})}}{1+e^{g(X_{i})}}=\frac{1}{1+e^{-g(X_{i})}},$$ (9)

often used in sequence. The mathematical simplicity of the logit function makes it the most used one.

Regression-based techniques represent to date the most frequently used approaches to data-driven modeling in radiotherapy. Regression link functions are typically sigmoidal to achieve the nonlinear dose–responses seen experimentally and advanced methods in AI that are able to handle nonlinear data complexity more readily are becoming increasingly popular due to superior prospective classification performance in many areas of medicine.^8^^,^^74^

The main problem that could arise with phenomenological multivariable models is that many different conceivable models can be developed to be consistent with the used data set. These can describe the present data accurately, but may later turn out to be inconsistent with new data sets.^75^

Overfitting can occur when a model is fitted to a dataset in such specific detail that the result loses its general validity for different datasets.^76^ Furthermore, multiple alternative phenomenological models exist and choosing which one and why is more appropriate can be challenging and not theoretically solid. This induces a model selection uncertainty or instability.^28^ More generally, it is challenging to guarantee how good the model predictions will be without making strong assumptions; models that rely on variations and relations of the training data cannot guarantee accuracy in subsequent datasets with a different statistical structure. Statistically, a correlation does not always imply causality. As such, it is important to note that phenomenological models are, therefore, not strictly guaranteed to describe causal relationships.

##### Kernel-based methods

As an alternative to regression-based methods, kernels can be applied to classification problems that depend on the deconvolution of nonlinear datasets. Kernels operations can be applied to generate classifiers such as hyperplanes that differentiate (classify) higher-dimensional datasets accordingly. In radiology, kernels are particularly useful given the nonlinear interaction of input data. Mechanistically, they attempt to maximize the distances between groups (clusters) and so kernels can be considered as extensions to Fischer linear discriminants or principal component analysis (PCA).^77^^,^^78^

PCA is a well-known approach used widely both within and outside of radiology and so further details can be found in previous works.^62^^,^^79^

Support-vector machines (SVMs) are the most often encountered kernel variant.^80^ An SVM seeks to redefine classification steps into maximization problems through the use of quadratic programming. Using such an approach, a computationally inexpensive SVM can be formulated as follows:

$$f(X)=\overset{n_{i}}{\underset{i=1}{\sum }}\alpha_{i}y_{i}K(S_{i},X)+a_{o},$$ (10)

wherein n is the number of support vectors, K is the kernel transformation, and $\alpha_{i}$ is the coefficient that is to be optimized by quadratic programing. SVMs are, therefore, nonparametric and are robust tools for higher-dimensional data classification.

### 6.2 Radiogenomics

In data-driven modeling, genetic parameters can be considered as independent variables and, for example, regressed alongside clinical risk factors and dose–volume metrics. For analytical models, genetic parameters can be integrated using different model-dependent methods.

#### 6.2.1 Augmented analytical models

Dose and volume-based TCP or NTCP models can be augmented by the integration of biological variables to enhance their prediction performance. The most frequently used example for this is the LKB model, which can be modified to include dichotomous clinical or genetic risk factors using $\delta_{k}$ as DMF

$$\mathrm{NTCP}(D,D_{50},m)=\frac{1}{\sqrt{2\pi }}\int_{-\infty }^{t}e^{\left(\frac{-u^{2}}{2}\right)}du,$$ (11)

$$t=\frac{D-TD_{50}(v)\cdot e^{\delta_{1}R_{1}}\cdot e^{\delta_{2}R_{2}}\cdot \ldots \cdot e^{\delta_{k}R_{k}}}{m\cdot TD_{50}(v)\cdot e^{\delta_{1}\cdot R_{1}}\cdot e^{\delta_{2}R_{2}}\cdot \ldots \cdot e^{\delta_{k}R_{k}}},$$ (12)

where $\delta_{k}$ is the weighted risk-factor coefficient for risk-factor k. The $R_{k}$ parameter is binary and indicates the presence ($R_{k}=1$) or absence ($R_{k}=0$) of the risk‐factor for a given patient.

In our previous work, we used a modified LKB model to integrate clinical risk factors together with SNPs and CNVs for predicting late effects of hypofractionated prostate radiotherapy.^27^ We found there to be benefit in the inclusion of select deoxyribose nucleic acid (DNA)-repair associated genetic mutations, but no clinical parameters improve the fit of either severe late rectal bleeding (RB) or late erectile dysfunction models. Similarly, a separate group demonstrated the value of including a clinical risk factor related to abominable surgery (yes/no) for the prediction of late RB toxicity in prostate cancer patients that underwent surgery.^81^

Imaging information can similarly be used as per the above examples. For example, in the context of patients with hepatocellular carcinoma having undergone liver irradiation, the addition of imaging information related to perfusion of their portal veins was found to improve the classification performance of the dosimetric LKB model for predicting severe (grade≥3) enzymatic changes.^82^ Such parallel approaches are often overlooked in favor of more complex modeling strategies, but are recommended in the first instance since they may be powerful yet robust. Indeed, understanding of the physiology at-hand is crucial for their coherence and it is possible that data-driven approaches may eventually lead to the identification of similar models but at the cost of time and effort.

While the above approaches have provided benefit in the context of statistical validation, we found the robustness of augmented analytical models to be reduced when compared directly with data-driven models of similar complexity.^27^

#### 6.2.2 Data-driven

Data-driven radiogenomic models function similarly to data-driven dose–volume models – see dose-volume-based approaches, data-driven for further details. They are most often based on regression (Fig. 1) so as to be able to handle almost any type of data, but kernel approaches can also be used. Regression frameworks allow stratification of risk factors through the use of dichotomous labels. For instance, the presence or absence of a genetic mutation for each patient can be incorporated into the input data as a 1 or 0, respectively.^83^ There is furthermore no limit to the number of such factors that can be included.

#### 6.2.3 Application of radiogenomics

Radiogenomic modeling consists of diverse approaches, making radiogenomic specific techniques difficult to define beyond the common integration of dosimetric, clinical, and biological data. We suggest to readers *a prior* work in which we directly compared the improvement in prospective classification achieved for predicting late side effects of prostate radiotherapy using dosimetry versus dosimetry plus common genetic risk factors.^27^ We also compared the improvement in regression-based models’ classification performance with those in quasianalytical models, such as the LKB, which thus provides an elegant introduction to the core principles of radiogenomics (dosimetric models, radiogenomic models, data-driven techniques, and analytical approaches).

An example of radiogenomics in the clinical setting is one that sought to identify predictive biomarkers of radiosensitivity in breast cancer patients toward classifying patients that are most likely to benefit from radiotherapy.^84^ The authors developed a signature for radiotherapy benefit based on two distinct biological processes: antigen processing/presentation (immune component) and intrinsic radiosensitivity. Using a validation cohort of 1439 patients, they found that radiation-sensitive patients who did not receive radiotherapy had a worse prognosis than otherwise. Patients in the immune-effective group that did receive radiotherapy had better disease-specific survival. To extend this study, the authors could have included treatment-related metrics in their analysis vis-à-vis radiotherapy treatment plan information; however, it is noted that there are relatively few critical structures adjacent to their target volumes and so may be unlikely to provide addition insight.

### 6.3 Machine Learning for Radiomics

Imaging-based approaches for outcome modeling typically take advantage of AI and ML techniques, which are able to mimic selected human behaviors to a degree, but must first be trained to be able to learn the patterns of interest.^61^^,^^85^ After training, such frameworks need to be tested on recognizing the pattern in a prospective setting (testing phase). Broadly speaking, there are two categories of frameworks that can be used: supervised (with labeled outcomes) and unsupervised (without labeled outcomes)—typically it is considered that validation is crucial for either approach but more so for the former.^86^ The use in oncology of conventional ML techniques, such as artificial neural networks (ANNs), has been successful^87^^–^^91^ but remains very much in its infancy in light of its potential.

#### 6.3.1 Conventional

Classical MI techniques in radiomics typically follow a top-down approach,^92^ where knowledge of interactions of radiation with tissue and biological systems is often ignored and radiotherapy outcomes are modeled through complex statistical analysis. For this reason, supervised learning would be preferred, i.e., models aimed at constructing a genotype–phenotype relationship by learning such genetic patterns from a labeled set of training examples. Nevertheless, an unsupervised approach (e.g., PCA or clustering) is sometimes used to reduce the dimensionality of datasets, extract a subset of relevant features, or construct features to be later included in the chosen learning method.^93^ The best way to model large datasets such as those containing genomic information is through support vector machines (SVMs).^80^ SVMs are tailored to identify a potentially complex unknown correlation structure between the phenotyping outcome and high-dimensional genotyping data.

Feedforward neural networks (FFNNs) are used frequently to train AI to basic pattern recognition problems. FFNNs do not include any recurring nodal inputs (memories) and are fully defined by their architecture such that arrangements of nodes into different topologies can induce different system responses. FFNNs have been used in radiation oncology due to their robustness.^94^^,^^95^ Typical activation functions are logit or probit functions, but also the radial basis function. Outputs from nodes are stratified by weights. Weights are the elements of the FFNN that are trained when building an FFNN. To adjust node input and output weights, the *delta rule* can be used via backpropagation until classification performance is optimized.^96^ Datasets for training can be used all at once (batch) or can be segregated into pattern-based subgroups (sequential).

#### 6.3.2 Deep learning

Deep learning can be contrasted with conventional learning by the fact that it does not require engineered features to be used as input to the framework.^97^ In other words, if found to be valuable for optimizing the objective, such inferences or associations can be synthetically derived by the framework itself and used to improve classification performance without declaring them *a priori*. In terms of probabilities, deep learning strategies can be categorized as either generative, whereby the joint probability (x,y) is used to estimate the posterior probability of (x,y), or as discriminative, wherein estimates of the posterior probability are made without calculating the joint distribution (direct mapping).

The decision to use one or the other is contingent upon the nature of the question (objective) of the framework itself. If the classification of data according to specific labels is all that is required, then discriminative approaches may be most relevant. However, if the dataset requires manipulation (e.g., is incomplete), then a generative approach would be indicated to manipulate the data into an acceptable form. Moreover, generative models, as the name suggests, allow higher-order parameters to be synthesized and used as input to the framework, or used to capture internal relationships of variables within the dataset. Extending the above to radiomics specifically, an example of discriminative learning may be the categorization of a volume as benign or malignant while the evaluation of intratumoural heterogeneity may warrant the use of generative strategies.

##### Discriminative deep learning

Convolutional neural networks (CNNs) are the most common type of network architecture in use today for image-intense processing,^98^ which is likely a consequence of increased accessibility to platforms that can generate such models (e.g., MATLAB^®^). They are used preferentially for the processing of especially large images.^99^ CNNs use local weights to control adjacent connections and then pooling to generate features that are spatially invariant. An important characteristic of CNNs is a fewer number of parameters that are trainable compared with an equal-sized ANN. Local filters can be used to further reduce the number of independent weights that are required. Transfer learning can be used to train a many-layered CNN to a rough approximation either to extract features or as a basis for an entire modeling process.^100^ As is the case with conventional learning, CNNs can make use of supervised or unsupervised methods (or a mix).

Another class of discriminative deep learning model is the autoencoder. Autoencoders are unsupervised neural networks that reconstruct underlying data through identification of intrinsic relationships between the input variables.^101^ Consequently, autoencoders reduce the dimensionality of input to a lower dimensional representation than is then input. When used alongside supervised frameworks, autoencoders offer a robust method to parse incomplete datasets. Similarly, encoders offer a way to resolve issues pertaining to sparseness when variance within training data is significantly increased compared with testing. Autoencoders can also be used in various architectures for classification or denoising of image data. We discuss in the next section a method for the use of autoencoders as generative models.

##### Generative deep learning

Variants of autoencoders can be used in the context of generative models (deep encoders) and have only recently found applications in feature extraction-based frameworks as well as for segmentation. The variational autoencoder consists of an autoencoder that produces latent vectors in a Gaussian distribution. The loss function includes both the Kullback–Leibler (KL) divergence between the latent vector and the Gaussian distribution as well as the squared error (mean) between the input and output. Other variants of deep autoencoders include those that make use of Bayesian methods for probability distribution that represents the data in question or convolutional autoencoders for the preservation of spatial locality, both of which are discussed in further detail in previous work.^102^

Another type of generative neural network is the deep belief network (DBN), which is several layers deep and consists of stochastic and latent variables.^103^ Every internal layer of a DBN serves as a hidden layer for the proceeding layer and as input to the successive layer. Consequently, a DBN can also be defined as an unsupervised rendition of a restricted Boltzman machine (RBM) or autoencoder. Importantly, DBNs are trained in a greedy manner using input from each previous layer. Relatedly, RBMs use both hidden and visible layers with forward passes learning the activation probability and backward passes the probabilities of inputs according to activations. In contrast to autoencoders, RBMs use stochastic units according to specific distributions rather than deterministic units. In other words, RBMs estimate joint probability distributions for inputs and activations and DBNs can, therefore, be defined as a stack of RBMs. In radiomics, labeled datapoints are often unavailable, and therefore, a combination of supervised and unsupervised techniques is often selected. For instance, a supervised network can be trained with the loss component estimated from unsupervised models such as RBMs.

Reinforcement learning (RL) is defined by an algorithm that seeks to maximize defined criteria leading to a reward given specific tradeoffs.^102^^,^^104^ The requirements for the use of RL in the context of deep learning include adequate knowledge of the environment, a defined reward, a defined value function, and governing policies (rules). In other words, RL approaches are goal oriented. For instance, RL approaches could be used to maximize the local control probability for a patient weighing the relevant risks of aberrant normal tissue damage. In this sense, RL learning provides an avenue for the integration of TCP and NTCP models. The use of RL in the context of deep learning has been used successfully to a degree for imaging-based landmark detection and, to a more limited degree, for radiotherapy treatment response predictions.^105^ While the case for the use of RL in the context of landmark detection in imaging is relatively straightforward provided one has an adequate training set, radiotherapy responses are an intrinsically more complex, multiscale phenomenon subject to inter- and intrapatient heterogeneity. Attempts to improve response predictions in radiomics, therefore, may necessitate the use of multiomics strategies, for which deep RL may provide benefit.

#### 6.3.3 Applications of radiomics

We briefly consider here three examples of radiomics applied clinically. The first example by Wu et al. sought to identify breast cancer subtypes.^106^ The authors retrospectively analyzed dynamic contrast-enhanced MRI data of breast cancer patients at their institution and extracted features from their dataset. Using an outside, multiinstitutional validation cohort, a gene expression-based classifier of imaging subtypes was developed and further tested against publicly available datasets. The result was a three-way classifier for stratifying recurrence-free survival of breast cancer patients. Indeed, in such a case, radiomic analysis would likely prove complementary to classical clinical histologic/molecular subtype categorization.

The second, more recent example by Jiang et al. developed a noninvasive radiomic signature predictive of gastric cancer ImmunoScore, which is a classifier of 27 immune cell features shown previously to effectively predict reoccurrence.^107^^,^^108^ Using four independent cohorts consisting of N=1778 patients, the authors extracted >500 quantitative features from contrast enhanced CT images. To correlate imaging features/metrics with the ImmunoScore results, a logistic regression model was then used. The result was a relatively robust signature of 13 imaging features that was found to strongly correlate with ImmunoScore, thereby providing a noninvasive surrogate marker.

The third example we discuss using radiomics incorporates deep learning for the prediction of survival in patients with glioblastoma.^109^ In this work, the authors used transfer learning to extract deep features and thereupon build signatures for survival. Using nearly 100,000 deep features alongside a handful of handcrafted features extracted from preoperative multimodality MR images, a six-deep-feature was constructed using least absolute shrinkage and selection operator (LASSO) Cox regression. The proposed signature was found to have significantly better classification performance of patients (prognostically). At the same time, the work demonstrated the feasibility of using transfer learning with deep features, a now frequently used technique for clinical radiomic studies.^110^^–^^112^

### 6.4 Panomics

**Fig. 3:**
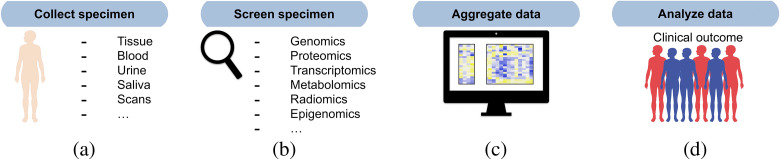
Workflow for panomics modeling. (a) Significant data can be generated in the clinical setting for patients undergoing radiotherapy through the careful analysis of specimens collected from certain sites and at certain times (pre/posttreatment). (b) Specimens can be used to generate data such as transcriptomics, genomics, proteomics, metabolomics, imaging correlates, and more. These datasets are usually high-dimensional and generated through NGS techniques. (c) Together with dosimetry and other clinical risk factors used in radiotherapy, datasets are preprocessed (normalization, filtering, for instance) and (d) applied to modeling of treatment outcomes (e.g., tumor response, toxicity).

The term panomics encompasses the use and integration of datasets relating to the multiomics technologies, such as genomics and proteomics,^12^ but also for our purposes here includes imaging correlates, clinical data, and data produced from other NGS platforms not specifically mentioned (Fig. 3). Given the heterogeneity of such datasets, their potential sparseness, and inconsistencies in their constituent data (missing values), working with them can pose unique challenges but also provide valuable insight. The favored approach for leveraging panomics datasets is, for now, one of systems biology.^113^

A systems biology approach recognizes that radiotherapy occurs at the intersection of physical processes and biological and clinical effects, each of which can take place over very different timescales. For instance, atomic-level effects of free radicals induced by ionizing radiation through to clinical scoring of late radiotherapy responses can span $10^{-9}$ to $10^{7}\;s$, for which the entirety of events are unlikely to adequately captured by any one single approach.^114^ Radiotherapy is also data intensive in that it encompasses functional imaging data and requires anatomical information, which, when coupled with biological markers from tumor and peripheral bloods, lends itself to the use of informatics approaches that can cope with high-dimensionality and heterogeneity.^115^

#### 6.4.1 Systems biology

The use of systems biology approaches for prediction of radiotherapy outcomes has to date been limited, but as discussed, remains a promising avenue for future works that make use of panomics datasets. Here, we review strategies for the integration of unique datasets but recognize that this list is by no means complete. In truth, the strategies adopted for working with panomics datasets are likely to vary to a degree between applications owing to the specific differences of each dataset at hand; however, several approaches described below can be used in the first instance.

##### Graph networks

In the case of the integration of proteomic and genetic data, graphical networks can be used wherein nodes represent genes/proteins and network edges their interactions. Such approaches make use of Bayesian networks, which our team has previously applied to the prediction of radiation-induced pneumonitis.

##### Similarity network fusion

The similarity network fusion (SNF) approach uses a network fusion method to integrate multiomics datasets (align datasets).^116^^,^^117^ In the first instance, an individual network is created for each dataset and then a nonlinear fusion network is used to combine them. The resulting network is coined a similarity network and the step by which the networks are combined (fusion step) uses belief propagation to accomplish this.^118^^,^^119^ The advantage of using an SNF approach is that noise can readily be identified as specific to each dataset and dissolves through the integration of the networks. On the other hand, true signals that are identified are propagated through the similarity network.

##### Joint Bayesian factor

This approach is nonparametric and uses joint Bayesian factors (JBFs) to integrate multiple data sets. Using this approach, the entire feature space is factorized into shared and unique components using a beta-Bernoulli process.^120^^,^^121^ The product is a joint factor model with two constituents: individual factors unique to each dataset and shared features, both of which can include noise (noise specific to a dataset and noise common to multiple datasets).^122^ Relative sparseness of each dataset can be evaluated and included in the analysis using a Student’s t-test sparseness factor. The advantage of using a JBF approach to integrate datasets is that it allows identification of a subset of factors that maximizes the overall objective function of the framework in an unbiased way.

##### Multiple coinertia analysis (MCIA)

MCIA attempts to capture relationships across high-dimensional datasets and has previously been used for gene expression and transcriptomics.^123^^,^^124^ One advantage of MCIA is that features do not need to necessarily be present for all subjects; however, the number of samples in each dataset must match. Covariance optimization criterion is used to transform the individual datasets and normalize them to the same scale. The datasets are projected onto the same unit space, which can then be searched. Applications of graph theory can facilitate this process and allow the extraction of heterogeneous features that are partial members of multiple datasets.^125^

#### 6.4.2 Challenges specific to panomics

The heterogeneity of large panomics datasets, their size, the requirement for intensive computational power, and also the lack of clarity as to the most appropriate methodology for their analysis makes working with such datasets challenging.^12^^,^^126^ Indeed, efforts have been made to resolve these challenges, but few have yet been widely adopted. For instance, the use of specific dataset formats for analysis is critical and *Feature X Sample* matrices have emerged as candidates for this purpose,^127^ but successive steps in processing panomics datasets remain unclear. Indeed, preprocessing and batch effect interrogation is paramount to the reliability of input data, and therefore, to the entire framework. Further processing prior to modeling includes normalization, filtering, and quality assurance, any one of which may perturb or obscure the underlying signal being sought. In a broader sense, panomic analyses must first strive to use the appropriate datasets and formats as they pertain to the question at hand. For example, it is straightforward to normalize datasets that originate from very different sources, but it is more difficult to take into account unequivocal outcomes or assay readouts.^128^ Moreover, interpretation of clinical information in the context of panomic datasets poses a unique challenge such that clinical metadata may be less easily quantified compared with NGS-generated data, both of which may change over time.

### 6.5 Evaluation of Model Performance

There is a diverse list of techniques that can be used to evaluate the performance of a model prior to independent validation on unseen data, which is typically the final step in a modeling framework. The techniques discussed herein are some of the most commonly employed methods but are by no means exhaustive. While they classically are applied to non-MI techniques, they can occasionally be found integrated into MI-based frameworks throughout radiology.^39^^,^^129^^,^^130^

#### 6.5.1 Estimation of error, optimal parameters, and model order

##### Statistical resampling

Resampling can be used to estimate optimal model order, best-fitting parameters, and/or error.^29^ Regardless of when it is used, statistical resampling divides a dataset into smaller sets in the first instance. When done with replacement, this can be repeated to produce a much larger pseudoset that can then be used to provide estimations of parameters without having to fully elucidate the true underlying distributions of the individual parameters themselves. The method is, therefore, nonparametric. Certain variations can be extremely quick as well, serving as easy-to-implement internal validations throughout the modeling process.

##### Jack-Knifing

Jack-knifing is a special case of cross-validation, whereby the first datapoint (e.g., patient) is left out and modeling is performed on the remaining cohort (N-1). The datapoint is then replaced and the second datapoint is removed and a new model is fit using all but the second datapoint. This is repeated until each successive datapoint is removed, generating a new model at each turn for a total of N. By examining the resulting models, insight into the robustness of the best-fit model obtained using all of the training data can then be analyzed. Jack-knifing is quick, but provides only an estimate of the more computationally intensive bootstrap technique. A more general version is to use k-fold cross validation.

##### Bootstrapping

Similar to jack-knifing, bootstrapping is a resampling technique done with replacement that can provide estimation of parameters, model orders, and errors. In contrast to jack-knifing, however, bootstrapping randomly resamples a select number of parameters. These subgroups can then be used to generate estimates of interest—a technique known as bagging. By analyzing their relative variability or averaging them, estimates for best-fit values can be produced. As a resampling technique, bootstrapping is nonparametric and especially finds use when analytical estimation of errors is not feasible. However, it should be noted that bootstrapping assumes independence of each datapoint from one another—this is usually not an issue for outcomes modeling given the independence of each patient from one another.

##### Applied information theory

In contrast to resampling techniques, information theory can be used to provide insight into the balance of model complexity and its fit. These approaches are quick but do not provide insight into the quality of a model nor of its interpretability.^131^

Akaike information criterion (AIC)

The AIC is a technique based on goodness of it and penalizes a model for underfitting or over-fitting

AIC=2k−ln(L), (13)

where the parameter k is the number of variables in a model and L is the log-likelihood. By using the KL distance to quantify the relative proximity between the two real and predicted probability distributions, an estimate of the divergence can be made. The optimal AIC value is found by minimizing the log-likelihood term on the right-hand side. AIC rewards models with fewer parameters that can adequately explain the data over those with increased complexity. The 2k is the penalty factor, which increases the AIC and whose contribution to the AIC cannot be overcome with better fit (lower log-likelihood). A drawback of the AIC is that it fails to perform adequately when k is large and numerous comparisons are being made. For instance, in the context of radiomics models that use NGS data, AIC would likely not to be suitable for the evaluation of goodness-of-fit.

Bayesian information criterion (BIC)

The BIC is a method similar to that of the AIC but differs in the arrangement of the constituent variables^132^ and furthermore includes a parameter n, which is the number of datapoints contained within the input data (sample size)

BIC=k·ln(n)−2·ln(L). (14)

The penalty term of the BIC is larger than that of the AIC and so penalizes overfitting more.

#### 6.5.2 Model performance evaluation

There exist numerous methods in literature to evaluate the ability of a given model to classify data in a prospective sense. Oftentimes, frameworks will employ more than one validation technique to explore the shortcomings of outputted models.

##### Validation coefficients and metrics

Metrics and coefficients are the most readily available tools for calculating the prediction or classification performance of outcome models. Their simplicity is amenable to quick understanding of model behavior and, when several are used together, can yield insightful information. The linear Pearson’s correlation is an example of a nonparametric coefficient that is used frequently for estimating the linearity of a relationship between two variables. More often employed in outcome models is the Spearman rank coefficient, which does not assume linearity and instead yields an estimate on the direction of trend between two parameters. Alternatively, receiver-operating characteristic (ROC) values can be summed from ROC plots to readily convey classification performance alongside sensitivity and specificity for the desired classification cut-off value. The selection of such cutpoints can be optimized using Youden indices, for instance.

##### Cross-validation by resampling

Resampling with replacement can be used to quantify the classification performance of models as well as estimate confidence intervals on model performance or provide estimates on the error of classification statistics. In our experience, leave-one-out cross-validation on finalized models serves as an excellent method to quickly estimate how robust a given model is without having to rely on more computationally expensive methods, such as bootstrapping.

## 7 Common Challenges and Pitfalls in Radiogenomics

Radiogenomics modeling requires the accurate processing, analysis, integration, and interpretation of large data sets, often simultaneously. Therefore, there are many challenges that need to be addressed to avoid errors and their propagation. Each set of data can involve a wide range of data types, including nominal, ordinal, and quantitative data. As a result, radiogenomic analysis demands efficient and objective dissection of image features (radiomics) so that relevant features and information can be extracted reliably and measured.^133^^,^^134^ Current challenges include AI interpretation often compared with the expert opinion and image interpretation.^135^ Therefore, the understanding and representation of each input data type is of great importance to be able to perform any accurate analysis.

### 7.1 Curse of Dimensionality

The curse of dimensionality refers to the difficulty associated with analyzing and characterizing large data sets where potential data space increases nonlinearly with the number of dimensions (exponentially increasing sparsity).^136^ This is a difficult to avoid cost associated with the ability of working with large datasets and can lead to the overgeneralization of results from the unjustified application of certain methods. For this reason, appropriate methods must be applied with judicious interpretation of the results. For example, the false discovery rate represents the expected rate of the inappropriately rejected null hypothesis (the expected proportion of false-positive results) and is frequently used in genomic analyses. The most commonly recognized challenge in the analysis of large data sets is the need for multiple hypothesis testing corrections, of which the Hochberg–Bonferroni P value correction is the simplest and perhaps the most well-known.^137^

The challenges of utilizing a large number of variables in outcome models are well summarized by the multiple testing dilemma: too few samples relative to a large number of variables being tested can lead to spurious correlations. Even after utilizing simple supervised learning algorithms to preprocess the data, the number of mRNA transcripts that a single microarray experiment can yield is often in the thousands.^81^ This issue can be mitigated by large-scale validation studies, but these are expensive, time-consuming, and patient accrual can limit achieving the necessary sample size.

Alternatively, methods in AI are becoming increasingly popular to explore the complex, hidden relationships between outcomes and biological variables.^82^ In contrast to brute-force estimating of correlations, machine-learning techniques have the ability to process highly structured, high-dimensional data while controlling for over- and underfitting by drawing on methods from control, probability, and information theory. Outside the applied field of radiomics, one group has provided a mathematical formulation to guide the detection of dimensionality anomalies as they are applied to deep learning and can be visited for further details.^138^

An important point is that neural networks and especially deep network learning strategies may be impervious to the curse of dimensionality by virtue of their architecture.^139^ Instead, they may suffer from challenges relating to dimensionality reduction during the data representation process.

### 7.2 Dimensionality Reduction

While complex methods are often needed by large data sets analysis, there is often a dangerous temptation to overfit the data to inordinately complex or overly parameterized models.^140^ The most common statistical tests assume normal distributions for measured variables. Despite this possibly being a fair assumption, nonparametric approaches can be used when the variables are being analyzed as an alternative. These include Bayesian-based approaches, which can prove particularly useful, given that clinical data are often complex and nonlinearly distributed. It is of paramount importance to note that just because a modeling calculation can be performed does not mean that it should be performed, and that underlying assumptions for use of the methods always need to be satisfied by the data to which they are being applied. We again note that validation is always the most important test to avoid under- or overfitting.

### 7.3 Data Preparation

Preprocessing of input data for radiomics frameworks can be critical, especially in the context of MI and panomics techniques.^141^ Data preparation can have a significant effect on the resulting classification result and so cannot be taken for granted. During this phase, several steps must be considered, which may include randomization, controls for under/oversampling, discretization of continuous data, scaling of features, and so on. As they pertain to all datasets, we focus our discussion here on randomization, feature scaling, and class balancing (sampling artifacts).

Features that are extracted from images during radiomic modeling can have very different scales, which, if not properly considered, may preclude interrogation of a subset of the feature space in any reasonable amount of time and furthermore risks destabilizing the framework. Specific examples of this include the weights in neural networks, which should be tuned to common scales across all nodes. By using a shared numerical scale, distortion of distributions through the networks can be avoided. It is, however, important to distinguish normalization from standardization. The former specifically refers to a scaling of between 0 and 1 while the latter is typically used to refer to Z-scoring of a dataset, that is, setting the mean to 0 and the standard deviation to 1. Ultimately, the choice of one or the other (or both) is highly context dependent and should be considered ahead of time.

Randomization is similarly important for modeling in radiomics since the overall performance of the algorithm may have local optimums that can be mistaken for global extrema. These instances are typically straightforward to overcome using pseudorandom seeding factors or start points for initiation. When coupled with normalization, the scales of parameters within a framework can be contained to within reasonable values. In the event that randomization techniques are not applied at the outset of modeling, certain motifs within the dataset may overwhelmingly influence the resulting model.

Class balancing is critical to ensure that the resulting model does not overtly misrepresent the signal of interest vis-à-vis under- or overfitting. Resampling techniques can resolve this challenge. In the context of radiogenomics, bootstrapping and jack-knifing provide quick and robust methods to achieve this, but for MI strategies, solutions can be computationally expensive and more complex. The preferred technique for class balancing with MI approaches is the synthetic minority oversampling technique (SMOTE). SMOTE generates subsets of underrepresented data with added variances to facilitate interrogation of what effect their increased representation may have on the model in question. A more recent version of SMOTE, coined adaptive synthetic sampling approach for imbalanced learning (ADASYN), also exists specifically for the learning from imbalanced datasets.^142^ In addition, generative adversarial network (GAN)-based approaches are becoming more widely used for generating synthetic compensations for such imbalances.

### 7.4 Roadblocks to Translation and Explainable Artificial Intelligence

A limitation in the ability to broadly apply radiomics/radiogenomics to the wider field of radiology/oncology is translatability. Without a comprehensive understanding of how and why an algorithm performs a classification, hesitation to adopt it as a standard approach is likely to be very real and enduring. Interpretability of deep learning algorithms in particular is often questioned and, indeed, many have shown how such networks can be tricked into misclassifying relatively straightforward cases. For radiomics, such misclassification could eventually be lethal. Oftentimes, deep learning strategies are referred to as black boxes; however, this is likely unhelpful and probably dissuades many seeking to understanding the reasoning behind certain decisions that are made. Some efforts to tackle this problem—that is, to open the black box—have, however, yielded some success in recent years. For instance, through techniques such as deconvolution networks, network inversions, and activation maximization, we increase our understanding of how relationships among variables are managed internally. Nonetheless, translatability of deep learning strategies remains a challenge and an area of active research may be critical for widespread adoption. From a different perspective, ML approaches can broadly be classified into interpretable and noninterpretable. ^135^ While techniques of the former are conducive to understanding, and therefore, adoption, their performance is often limited, necessitating the use of the more abstruse (noninterpretable) methods. One of the most active cores researching explainable artificial intelligence (XAI) is at the Defense Advanced Research Projects Agency (DARPA).^143^^–^^145^ In this context, explainable rather than interpretable AI was chosen to highlight their desire to maximize human interactivity of their platforms. Medical XAI (mXAI) is a more recent development with a similar objective of opening the black box.^146^^,^^147^ In turn, mXAI seeks to provide discrete, high-level accountability, and therefore, transparency of deep learning algorithms in the medical sciences.

### 7.5 Rare Variants Role

The inclusion of biological risk factors can generate challenges for outcome modeling, regardless of their value for classification performance.^148^ In the case of variants that are uncommon across a dataset but valuable for classification purposes, an imbalance can manifest itself during the modeling process if identified as important factors during the modeling process. This may be especially true when dichotomous biological risk factors are included within a framework as they risk overperturbing the real increase in risk for the variant (0% or 100% additional risk). By definition, deleterious genetic mutations are rare variants when compared with the much larger number of noncoding and innocuous mutations. The role of rare variants is particularly challenging to study because MI approaches are often used to try to identify them, but they would ideally be known *a priori* and compensated for once the relevant pattern has been identified (chicken and egg). Currently, no techniques are widely adopted to take into account challenges associated with rare variants.

### 7.6 Echo Chamber Effect

The echo chamber effect occurs when working with large datasets, such as for radiomics, when an internal relationship in the data is cyclically amplified through the process of data aggregation and is, therefore, considered a variation of selection bias such that the training dataset is not representative of the population.^149^^,^^150^ An example is a meta-analysis, which, when performed in the context of biomarkers, has an implicitly lower probability of a negative result and thus deviates from real-world conditions.^151^

The opposite of the echo chamber effect is the Yule-Simpson paradox whereby true associations are identified and reported in smaller cohorts, but are lost in larger datasets.^152^ Example and issuing controversies from real-life instances of both the above are described in our previous work.^149^

### 7.7 Data Injustices (Bias and Mitigation)

Biased decision making is not a trait inherently unique to MI nor to radiomics, but is none the less a real risk.^153^ Dissecting the origin of biases that are observed in testing and validation is likely to point toward unrepresentative biases within the training dataset itself. Unfortunately, as a result, cross-validation techniques and resampling methods discussed earlier cannot overcome these hurdles and independent validation on unseen data is usually required. Within and outside the field of radiomics, these training data biases can occur due to geography of cohort data or any other number of ways, but similarly converge to produce suboptimal models, especially in the context of learning algorithms with memory. Injustices within datasets differ from small sample sizes by passing internal validation and potentially even on unseen data. Rather, they are more cryptic, and therefore, more challenging to resolve. For example, geographical biases may overexpose a training dataset toward specific ethnic or racial backgrounds, and therefore, be unapplicable on a wider population. These challenges can similarly be institutional or social. One method to overcome this challenge is the annotation of the methodology used for the aggregation of datasets in questions. This produces yet another challenge for standardization of, for example, experimental protocols, and may be challenging if identifiable patient metrics are necessitated. An alternative strategy is the use of dedicated MI algorithms to seek out biases unknown to operators. This could either be interpreted or fed directly into the core modeling framework to systemically audit the underlying strategy and maximize its applicability.

## 8 Conclusion and Perspectives

In this work, we discuss two complementary but distinct strategies for predicting radiotherapy outcomes: radiogenomics and radiomics. Both techniques take advantage of biological variables to augment dosimetric risk factors; however, radiogenomics entails the integration of biological, dosimetric, and clinical factors whereas we considered radiomics to further include the use of imaging correlates. As discussed, radiomics quickly necessitates the use of MI strategies for implementation since it is not evident which textures or features may be relevant to a given outcome (nor even in which dimensions they reside). Conversely, radiogenomics consists of a more diverse set of strategies, some more straightforward than others. For this reason, it is often practical to first familiarize oneself with techniques for radiogenomic modeling before generating and integrating imaging correlates for use alongside biological data in predicting radiotherapy outcomes.

Going forward, we feel it is likely that these two techniques (radiomics and radiogenomics) will intersect, but for this to occur will necessitate the use of high-throughput amenable methods—high dimensional datasets from diverse sources can provide unique insight to a broad patient population but often present a multitude of challenging hurdles at the analysis stage. Thus, we expect that clinically viable predictive frameworks for radiotherapy will trend toward panomics strategies, which are designed to shift through highly heterogeneous/incomplete datasets inclusive of imaging correlates and multiscale biological data. It is our opinion that the noninvasiveness of imaging and the potential capabilities of next-generation platforms together with high spatial resolution will continue to motivate and support the use of radiomics/panomics going forward.

More broadly, we expect that AI and MI techniques will overhaul most aspects of radiation oncology, from delivery, treatment planning, and optimization to outcomes. The integration of such strategies will necessitate further training for physicians and physicists alike as well as development of quality assurance strategies and culpability schemas. Given the complexity of such modeling frameworks, it is possible that poorly trained personal or avoidable misinterpretations may do more harm than good. Thus, continuing to draft and refine policy for potential clinical pipelines using radiomics/panomics is critical. In the interim, we expect imaging biomarkers to continue to be useful for diagnosis and treatment.
